# Comparative mitogenome research revealed the phylogenetics and evolution of the superfamily Tenebrionoidea (Coleoptera: Polyphage)

**DOI:** 10.1002/ece3.11520

**Published:** 2024-06-25

**Authors:** Yun‐Jian Hu, Feng‐Fan Jia, Li Hu, Chuan Wu, Tian Tian, Ting‐Jing Li, Bin Chen

**Affiliations:** ^1^ Chongqing Key Laboratory of Vector Insects, Institute of Entomology and Molecular Biology, College of Life Sciences Chongqing Normal University Chongqing China

**Keywords:** Coleoptera, evolution, mtgenome, phylogenetics, Tenebrionoidea

## Abstract

Despite the worldwide distribution and rich diversity of the superfamily Tenebrionoidea, the knowledge of the mitochondrial genomes (mtgenome) characteristics of the superfamily is still very limited, and its phylogenetics and evolution remain unresolved. In the present study, we newly sequenced mtgenomes from 19 species belonging to Tenebrionoidea, and a total of 90 mitochondrial genomes from 16 families of Tenebrionoidea were used for phylogenetic analysis. There exist 37 genes for all 82 species of complete mtgenomes of 16 families investigated, and their characteristics are identical as reported mtgenomes of other Tenebrionoids. The Ka/Ks analysis suggests that all 13 PCGs have undergone a strong purifying selection. The phylogenetic analysis suggests the monophyly of Mordellidae, Meloidae, Oedemeridae, Pyrochroidae, Salpingidae, Scraptiidae, Lagriidae, and Tenebrionidae, and the Mordellidae is close to the Ripiphoridae. The “Tenebrionidae clade” and “Meloidae clade” are monophyletic, and both of them are sister groups. In the “Meloidae clade,” Meloidae is close to Anthicidae. In the “Tenebrionidae clade,” the family Lagriidae and Tenebrionidae are sister groups. The divergence time analysis suggests that Tenebrionoidea originated in the late Jurassic, Meloidae Mordellidae, Lagriidae, and Tenebrionidae in the Cretaceous, Oedemeridae in Paleogene. The work lays a base for the study of mtgenome, phylogenetics, and evolution of the superfamily Tenebrionoidea.

## Graphical abstract

In the present study, we newly sequenced mtgenomes from 19 species belonging to Tenebrionoidea, and a total of 90 mitochondrial genomes from 16 families of Tenebrionoidea were used for phylogenetic analysis. The divergence time analysis suggests that Tenebrionoidea originated in the late Jurassic, Meloidae Mordellidae, Lagriidae, and Tenebrionidae in the Cretaceous, Oedemeridae in Paleogene.

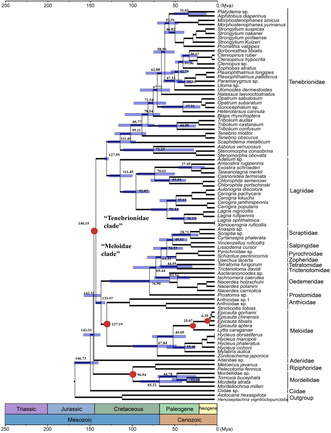

## 1 INTRODUCTION

Tenebrionoidea is a large superfamily in Coleoptera, with over 34,000 known species and 28 families (Lawrence, 1995; Ślipiński et al., 2011). The species in the superfamily have the 5‐5‐4 tarsal formula in both sexes, with occasional 4‐4‐4, 3‐3‐3 or 3‐4‐4 in males. Members of the superfamily demonstrate various types of feeding strategies, the majority of which are fungivorous, xylophagous, and saprophagous. Some Tenebrionoidea species are major agricultural and forest pests that attack commercial crops or stored products (Song et al., 2018). They are widely spread throughout all terrestrial habitats from the sea shore up to dry desert and steppe habitats in all altitudinal belts and all types of forests, and species in arid environments are conspicuously diverse.

Under the combined influence of various factors, including feeding strategies, habitats, cryptic speciation, developmental processes, sexual selection, convergent evolution, and other mechanisms, the morphology of the superfamily is complex (López‐Estrada et al., 2019, 2022). The species of Tenebrionoidea are of significant ecological and economic significance. For instance, the larvae of *Epicauta gorhami* from the Meloidae family feed on grasshopper eggs, whereas the adults take soybeans as food (Shintani et al., 2011), functioning as pest predator as well as pest in the ecological system. In addition, they produce a highly toxic substance called cantharidin, which is valuable for treating certain types of tumors (Zha et al., 2017). The *Tenebrio molitor* in Tenebrionidae, as a natural decomposer, has strong and rapid reproduction ability and ecological adaptability and provides food for a wide variety of birds and mammals in the field as well as are industrially bred to produce animal and even human food (Grau et al., 2017).

Taxonomic and phylogenetic studies are essential for understanding their biodiversity and biological characteristics and for further protecting, utilizing, and controlling them. The framework of the classification system for Coleoptera was firstly established in 1955 and then revised in 1982, in which the Tenebrionoidea is divided into five major lineages: (1) Tetratomidae, Melandryidae, Mordellidae, Ripiphoridae; (2) Synchroidae, Zopheridae, Prostomidae, Perimylopidae, Chalcodryidae, Tenebrionidae; (3) Oedemeridae, Stenotrachelidae, Meloidae; (4) Pythidae, Pyrochroidae, Boridae, Mycteridae, Salpingidae; and (5) Anthicidae, Aderidae, Scraptiidae (Crowson, 1955; Lawrence & Newton Jr, 1982). In past years, a number of studies have been reported to establish the taxonomy system of Tenebrionoidea using comparative morphology data (Beutel & Friedrich, 2005; Lawrence et al., 2011). Nowadays, 28 families (Mordellidae, Ripiphoridae, Anthicidae, Meloidae, Pyrochroidae, Oedemeridae, Salpingidae, Scraptiidae, Lagriidae, Tenebrionidae, and so on) are widely recognized for Tenebrionoidea (Bouchard et al., 2011). Despite its economic importance, the monophyletic status of many families and phylogenetic relationships in Tenebrionoidea need to be elucidated. A comprehensive phylogeny study of beetles showed that the two families Mordellidae and Meloidae are monophyletic, while the Scraptiidae is paraphyletic based on *18S rRNA*, *16S rRNA*, and *COX1* gene sequences (Hunt et al., 2007). The phylogenetic analysis of Coleoptera showed that the families Tenebrionidae, Oedemeridae, Salpingidae, Mordellidae, Meloidae, and Scraptiidae are monophyletic; Pyrochroidae is polyphyletic based on four gene (*16S rRNA*, *COX1*, *28S*, and *18S*) sequences (Bocak et al., 2014). The higher‐level phylogenetic analysis of beetles based on 95 nuclear protein‐coding genes from 373 beetle species proposed that the Tenebrionidae, Oedemeridae, Salpingidae, Pyrochroidae, Meloidae, and Scraptiidae were monophyletic, whereas Anthicidae was paraphyletic (Zhang et al., 2018). The study based on 1,907,014 amino acid sites from 4818 nuclear genes across 146 beetle species proposed that Tenebrionidae, Ciidae, Ischaliidae, Salpingidae, Zopheridae, Stenotrachelidae, Scraptiidae, Prostomidae, Trictenotomidae, Mycetophagidae, Oedemeridae, Aderidae, Pyrochroidae, Chalcodryidae, Tetratomidae, Tentratomidae, Synchroidae, Archeocrypticidae, and Pterogeniidae were monophyletic. In the later study, based on the extraction of 68 single‐copy nuclear protein‐coding (NPC) genes from 129 families suggested that the Oedemeridae, Salpingidae, Pyrochroidae, Meloidae, Zopheridae, Ciidae, Ulodidae, Mycetophagidae, and Tenebrionidae were monophyletic (Cai et al., 2022). Almost all molecular phylogenetic analyses suggested the monophyly of Mordellidae and Meloidae; however, it is inconsistent with the results based on morphological data (Lawrence et al., 2011). Due to incongruent phylogenetic relationships from different analyses, the phylogenetic relationships and monophyly among families need to be elucidated.

With the characteristics of conservative gene content, simple genome organization, maternal inheritance, small genome size, and higher evolution rate, the complete mitochondrial genome (mtgenome) has been widely used in molecular phylogenetics, evolution, and population genetics study of insects (Cameron, 2014). Up to date (October 2021), there have been 71 species of Tenebrionoidea complete mtgenomes to be reported in GenBank, covering 15 families in Tenebrionoidea. However, there is no complete mtgenome sequence to be reported from the families Archeocrypticidae, Stenotrachelidae, and Melandryidae, and the characteristics of the mtgenomes are still little understood in the superfamily. Up to now, there have been only a few mtgenome‐based phylogenetic studies at high taxa in Tenebrionoidea. Phylogenetic inference of beetles indicated that the families Tenebrionidae, Oedemeridae, Mordellidae, and Meloidae were monophyletic, whereas Scarptiidae was polyphyletic based on 245 mitochondrial sequences using Bayesian method (Timmermans et al., 2015). The phylogenetic analysis of 37 beetle mitochondrial genes supported the Mordellidae and Meloidae to be monophyletic groups and the Scraptiidae and Melandryidae to be paraphyletic groups using Bayesian inference (Song et al., 2018). The mtgenome‐based phylogeny of 51 beetles found the monophyletic of Meloidae, Tenebrionidae, and the sister relationship of Meloidae and Tenebrionidae using Bayesian inference (Tang et al., 2020). As mentioned above, previous mtgenome‐based phylogenetic studies have not well resolved the phylogeny of Tenebrionoidea, and phylogenetic analysis of the superfamily needs to be further evaluated.

In this study, we newly sequenced and annotated mitogenomes from 19 species belonging to Tenebrionoidea and comparatively analyzed the mtgenome characteristics of a total of 90 species in Tenebrionoidea. More importantly, we constructed and discussed the phylogenetic relationships at high taxa level based on these mtgenome sequences and inferred the divergence time at main nodes in the phylogenetics in the inference of fossil records. This study lays a foundation for further understanding of mtgenome characteristics, phylogenetics, and evolution of the Tenebrionoidea.

## 2 MATERIALS AND METHODS

### 2.1 Sample collection, sequencing, and mtgenome assembly

All samples were collected in China (Table S1) and then stored in 95% alcohol at −20°C until DNA extraction. These samples were identified in morphology, 19 species were selected for sequencing, and their identification was confirmed by subsequent *COX1* comparison with BOLD (http://boldsystems.org/index.php; Hebert et al., 2003). The total genomic DNA was extracted from the thorax and leg muscle tissue by DNeasy Blood and Tissue kit (Qiagen, Duesseldorf, Germany) according to the manufacturer's instructions. Concentration of extracted genomic DNA was determined by Qubit 2.0 (Invitrogen, Shanghai, China). The 350 bp small fragment libraries were constructed and then sequenced using the Illumina Hiseq 2500 (San Diego, CA) with 150 bp paired‐end reads in Shenzhen Huitong Biotechnology Co. Ltd (Shenzhen, China). After removing the adapters and unpaired, short‐ and low‐qualitied reads, clean reads from mtgenomes were extracted using a BLAST (Altschul et al., 1990) search against known Tenebrionoidea mtgenome sequences and then used for de novo mtgenome assembly with SPAdes v. 3.9.0 (Bankevich et al., 2012). The contigs of mtgenome were extracted and assembled into mtgenomes by searching against the reference sequences using PRICE (paired‐read iterative contig extension) by NOVOPlasty version 2.6.2 (Dierckxsens et al., 2016).

### 2.2 Mtgenome annotation and characteristics analysis

In the present study, a total of 90 species of mtgenomes in 16 families belonging to Tenebrionoidea as ingroup and two species in Coccinellidae as outgroup were subject to mtgenome annotation, and characteristics and phylogeny analysis, in which 71 species in Tenebrionoidea and two species in Coccinellidae were selected and downloaded from GenBank in October 2021. The rough annotation of protein‐coding genes (PCGs), transfer RNA genes (tRNAs), ribosomal RNA genes (rRNAs), and CR was initially identified using MITOS (http://mitos.bioinf.uni‐leipzig.de/index.py; Bernt et al., 2013) and then determined in comparison of published homologous mtgenome sequences in phylogeny‐close species using MEGAX (Kumar et al., 2018). The tRNAs secondary structures were predicted using tRNAscan‐SE Search Server v. 1.21 (http://lowelab.ucsc.edu/tRNAscan‐SE/; Lowe & Eddy, 1997). The annotation of the mtgenomes was corrected manually using Geneious v. 4.8.5 (Kearse et al., 2012), and final mtgenomes were submitted to the GenBank database. The secondary structures of the tRNAs were visualized and manually edited using VARNA (http://varna.lri.fr; Darty et al., 2009). The mtgenomes were visualized using the Chloroplot online server with default parameters (Zheng et al., 2020). Base composition and relative synonymous codon usage (RSCU) of 90 species of mtgenome were computed with PhyloSuite desktop platform (Zhang et al., 2020). AT‐skew [(A − T)/(A + T)] and GC‐skew [(G − C)/(G + C)] were estimated to investigate nucleotide composition bias (Perna & Kocher, 1995), and three‐dimensional scatterplots of AT‐Skew, GC‐Skew, and AT% were drawn using Origin Pro v. 9.0 (Mikrajuddin & Khairurrijal, 2009). The selection pressure of the 13 PCGs was analyzed by calculating Ka (nonsynonymous mutation rates) and Ks (synonymous mutation rates) values with DnaSP v. 5.0 (Librado & Rozas, 2009), and visualized using RStudio. Sequence saturation was assessed in DAMBE v. 5.0 (Xia, 2013).

### 2.3 Phylogenetic analysis of Tenebrionoidea

Phylogenetic relationships of 90 species of mtgenomes (including 19 sequenced in this study) in Tenebrionoidea were deduced using three data sets and two inference methods with *Aiolocaria hexaspilota* and *Henosepilachna vigintioctopunctata* (Coleoptera: Coccinelloidea) as outgroups. Taxonomic information for each species investigated and mtgenome accession numbers are listed in Table 1. Three data sets were concatenated using PhyloSuite platform: (1) amino acid sequence of 13 PCGs (AA), (2) nucleotide sequence at first and second codon position of 13 PCGs (PCG12), (3) PCG12 + 2 rRNAs, respectively, with excluding start codon, stop codon. Nucleotide sequences of 13 PCGs were aligned by codon‐based multiple alignments using the L‐INS‐i algorithm, and the rRNAs were aligned using the Q‐INS‐i strategy in MAFFT v. 7.0 (Katoh & Standley, 2013), and ambiguously aligned positions were excluded using Gblocks (Talavera & Castresana, 2007). The concatenation of aligned sequences was performed using SequenceMatrix (Vaidya et al., 2011). The selection of best‐fit partitioning schemes and substitution models for each data set were calculated using PartitionFinder v. 2.0 (Lanfear et al., 2016) with the settings: branch lengths as linked, model election as AICc with the greedy algorithm. Partitioning schemes and models are listed in Table S2. Two methods, maximum likelihood (ML) and Bayesian inference (BI), were employed for the deduce. ML‐based phylogenetic analyses were conducted using IQ‐TREE v. 1.6.8 in PhyloSuite v. 1.2.2 (Zhang et al., 2020). Nodal support values were inferred with 1000 bootstrapped replicates (BPs) (Minh et al., 2013). BI analysis was conducted using MrBayes v. 3.2.6 (Ronquist et al., 2012). A total of 2,000,000 generations with four chains were sampled every 1000 generations. Posterior probabilities (PPs) were computed after discarding the first 25% of trees as the burn‐in phase. The estimated sample size (ESS) >200 and the average deviation of the split frequency of less than 0.01 indicates that the runs had converged. The phylogenetic tree was visualized using FigTree v. 1.4.4 and iTOL online tool (Letunic & Bork, 2016).

**TABLE 1:** Taxonomic information and GenBank accession numbers for 90 mtgenomes in Tenebrionoidea selected for characteristics and phylogenetics analysis in this study.

Family	Subfamily	Species	Accession number	References
Tenebrionidae	Pimeliinae	*Asbolus verrucosus*	NC_027256	Rider (2016)
		*Stenomorpha obovata*	MZ342786	Smith et al. (2021)
		*S. consobrina*	MZ342785	Smith et al. (2021)
	Diaperinae	*Platydema* sp.	JX412842	Unpublished
		*Scaphidema metallicum*	KX087341	Unpublished
		*Ulomoides dermestoides*	NC_025332	Wang et al. (2016)
	Stenochiinae	*Strongylium Kulzeri*	CT5	This study
		*S. pinfaense*	CT6	This study
		*S. suspicax*	JX412780	Unpublished
		*S. nakanei*	MW802591	This study
		*Promethis valgipes*	NC_054362	Direct Submission
		*Morphostenophanes yunnanu*	MZ298928	Smith et al. (2021)
		*M. sinicus*	MW853764	Direct Submission
	Tenebrioninae	*Uloma* sp.	KT876915	Linard et al. (2016)
		*Nalassus laevioctostriatus*	KT876905	Linard et al. (2016)
		*Tribolium castaneum*	NC_003081	Friedrich et al. (2003)
		*T. audax*	NC_024600	Ou et al. (2016)
		*T. confusum*	NC_026702	Ou et al. (2016)
		*Tenebrio molitor*	NC_024633	Li‐Na et al. (2014)
		*T. obscurus*	NC_037196	Bai et al. (2018)
		*Zophobas atratus*	NC_041101	Bai et al. (2018)
		*Plesiophthalmus longipes*	S20	This study
		*Paramarygmus* sp.	JX412808	Unpublished
		*P. pallidicrus*	CT8	This study
		*Heterotarsus carinula*	CT9	This study
		*Gonocephalum* sp.	NC_053250	Direct Submission
		*Gonocephalum kochi*	MW822744	Unpublished
		*Blaps rhynchoptera*	NC_047449	Zhao et al. (2019)
		*Alphitobius diaperinus*	NC_049092	Hong et al. (2020)
		*Opatrum subaratum*	CT11	This study
		*O. sabulosum*	MN745102	Feng et al. (2020)
	Alleculinae	*Borboresthes tibialis*	CT13	This study
		*Cteniopus* sp.	KX087267	Unpublished
		*Cteniopinus ruber*	S21	This study
		*C. hypocrita*	CT14	This study
Lagriidae	Adeliinae	*Adelium* sp.	NC_013554	Sheffield et al. (2008)
	Lagriinae	*Aulonogria discolora*	CT41	This study
		*Lagria ophthalmica*	CT20	This study
		*L. nigricollis*	CT25	This study
		*L. rufipennis*	CT23	This study
		*Cerogria kikuchii*	CT24	This study
		*C. popularis*	S15	This study
		*C. janthinipennis*	CT28	This study
		*C. pachycera*	CT22	This study
		*Xenocerogria ruficollis*	CT32	This study
	Statirinae	*Chlorophila portschinski*	CT33	This study
		*C. semenowi*	CT34	This study
		*Anisostira rugipennis*	CT35	This study
		*Exostira schroederi*	MW802590	This study
		*Taiwanolagria merkli*	CT37	This study
		*Casnonidea terminata*	CT38	This study
Meloidae	Nemognathinae	*Zonitoschema japonica*	S18	This study
	Meloinae	*Epicauta gorhami*	NC_036042	Du et al. (2017)
		*E. chinensis*	NC_029192	Du et al. (2017)
		*E. tibialis*	NC_036043	Du et al. (2017)
		*E. aptera*	NC_031820	Jie et al. (2016)
		*Hycleus marcipoli*	NC_036044	Du et al. (2017)
		*H. phaleratus*	NC_036045	Du et al. (2017)
		*H. dorsetiferus*	CT18	This study
		*H. cichorii*	NC_039657	Unpublished
		*Lytta caraganae*	NC_033339	Yuan et al. (2017)
		*Mylabris aulica*	NC_036046	Du et al. (2017)
		*M. calida*	NC_050854	Unpublished
Oedemeridae	Oedemerinae	*Nacerdes potanini*	S44	This study
		*N. carniolica*	KX087319	Unpublished
		*N. holzschuhi*	CT1	This study
		*Ischnomera caerulea*	JX412790	Unpublished
		*Ascleranoncodes* sp.	CT4	This study
Scraptiidae	Scraptiinae	*Scraptia* sp.	JX412825	Unpublished
		*Cyrtanaspis phalerata*	KX087279	Unpublished
	Anaspidinae	*Anaspis* sp.	JX412856	Unpublished
Mordellidae	Mordellinae	*Mordella atrata*	NC_013254	Cameron et al. (2009)
		*Tomoxia bucephala*	KX087355	Unpublished
		Mordellidae sp.	JX412844	Unpublished
		*Mordellochroa milleri*	KX087318	Unpublished
Zopheridae	Zopherinae	*Usechus lacerta voucher BMNH 679350*	NC_036269	Linard et al. (2017)
Ripiphoridae	Pelecotominae	*Pelecotoma fennica*	NC_036277	Linard et al. (2017)
		*Metoecus javanus*	MZ618348	Unpublished
Ciidae	Ciinae	Ciidae sp.	JX412846	Unpublished
Tetratomidae	Tetratominae	*Tetratoma fungorum*	NC_036276	Linard et al. (2017)
Aderidae		Aderidae sp.	JX412763	Unpublished
Anthicidae	Anthicinae	*Stricticollis tobias*	KX087350	Unpublished
		Anthicidae sp.1	MH789722	Crampton‐Platt et al. (2018)
		Anthicidae sp.2	MG253255	Unpublished
Pyrochroidae	Pyrochroinae	*Schizotus pectinicornis*	KX087342	Unpublished
		*Pyrochroa* sp.1	S43	This study
Salpingidae	Salpinginae	*Vincenzellus ruficollis*	NC_036274	Linard et al. (2017)
		*Lissodema cursor*	KX087307	Unpublished
Prostomidae		*Prostomis* sp.	JX412787	Unpublished
Trictenotomidae		*Trictenotoma davidi*	MW580860	Sheng et al. (2021)
Outgroup (Coccinellidae)		*Aiolocaria hexaspilota*	NC_042417	Seo et al. (2019)
		*Henosepilachna vigintioctopunctata*	NC_041172	Zhang et al. (2019)

### 2.4 Divergence time estimation

Divergence time was estimated using the uncorrelated relaxed clock model as implemented in BEAST v. 1.6.1 (Drummond & Rambaut, 2007). In order to limit the numbers of parameter for the estimation, Bayesian tree was used as a guide tree. The tree prior generated using a Yule speciation model, and all node calibrations were enforced using Normal distributions. Constraints on clade ages were enforced using five fossil calibrations: *Protoripidius burmiticus* in Mordellidae (m = 96.55, SD = 2.0, offset = 0) diverged 93.5–99.6 Mya (Cai et al., 2018), *Camelomorpha longicervix* in Anthicidae–Meloidae (m = 127.75, SD = 1.25, offset = 0) 125.45–130 Mya (Kirejtshuk & Azar, 2008), *Alphitopsis initialis* in Tenebrionidae–Lagriidae (m = 127.75, SD = 1.25, offset = 0) 125.45–130 Mya (Kirejtshuk et al., 2012), *Meloe dominucanus* in *Lytta* (logm = 10, SD = 1.25, offset = 15) 50–15 Mya (López‐Estrada et al., 2022; Poinar et al, 2009), and *Epicauta sanctoruensis* in *Epicauta* (m = 4.47, SD = 0.5, offset = 0) 5.33–3.6 Mya (Caballero & de León, 2003). The posterior time estimation was conducted using an MCMC algorithm, and the MCMC run was sampled every 1000 iterations until it achieved 10,000 samples, after the first 100,000 iterations were discarded as burn‐in. The effective sample sizes of every node age were confirmed using Tracer v. 1.5 until every parameter was >200. The maximum clade credibility tree was calculated using TreeAnnotator v. 1.6.1, with the node times scaled to match the mean posterior estimates.

## 3 RESULTS

### 3.1 Mtgenome organization

**FIGURE 1:**
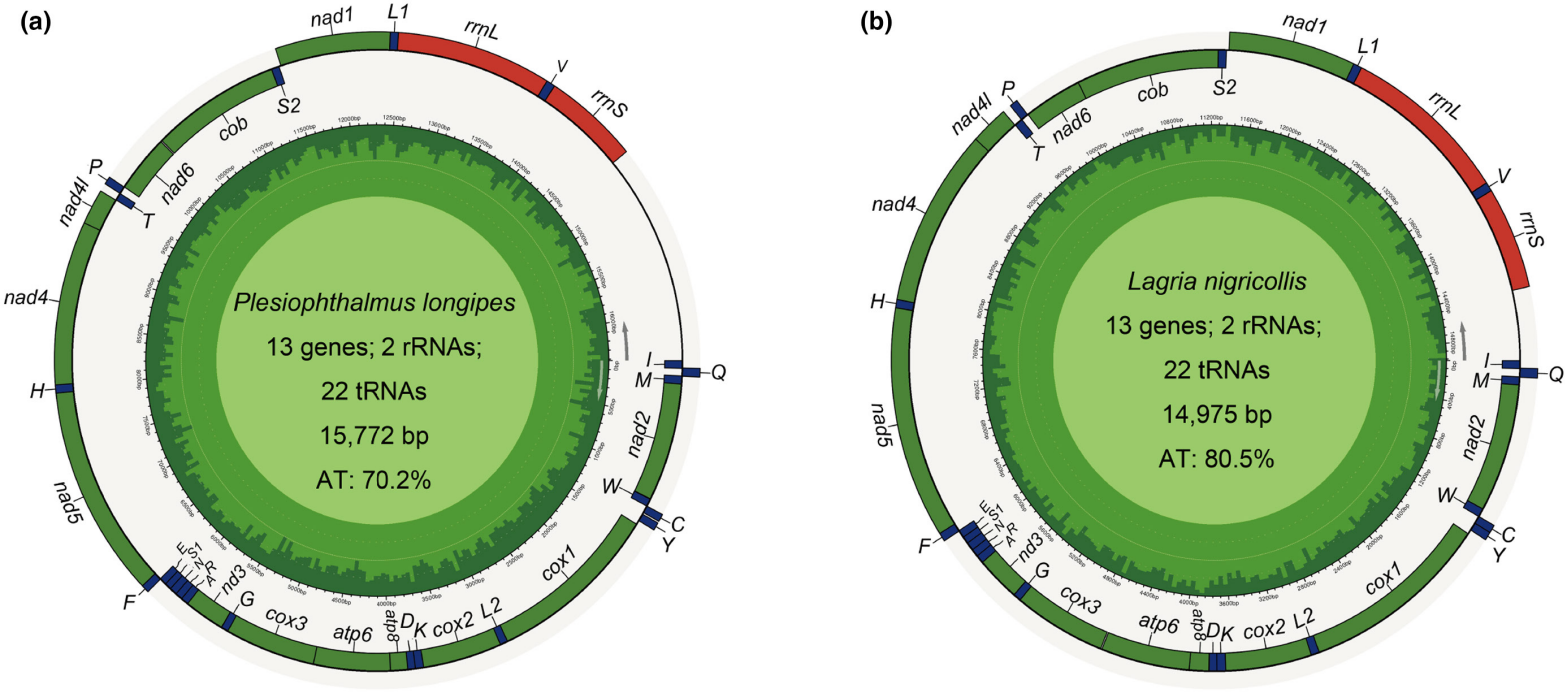
Complete mtgenome genome structure of *Plesiophthalmus longipes* (a) and *Lagria nigricollis* (b). The species name and specific information regarding the genome (length, AT content, and the number of genes) are depicted in the center of the figures. The gene names are labeled on the outer circle, the green‐, blue‐, and red‐filled blocks indicate PCGs, tRNAs, and rRNAs, respectively. The genes marked outward on the outer circle are located on the N‐strand, whereas the genes inward are located on the J‐strand. L1, L2, S1, and S2 represent the *tRNA‐Leu* (UAA), *tRNA‐Leu* (UAG), *tRNA‐Ser* (AGN) and *tRNA‐Ser* (UCN), respectively.

**FIGURE 2:**
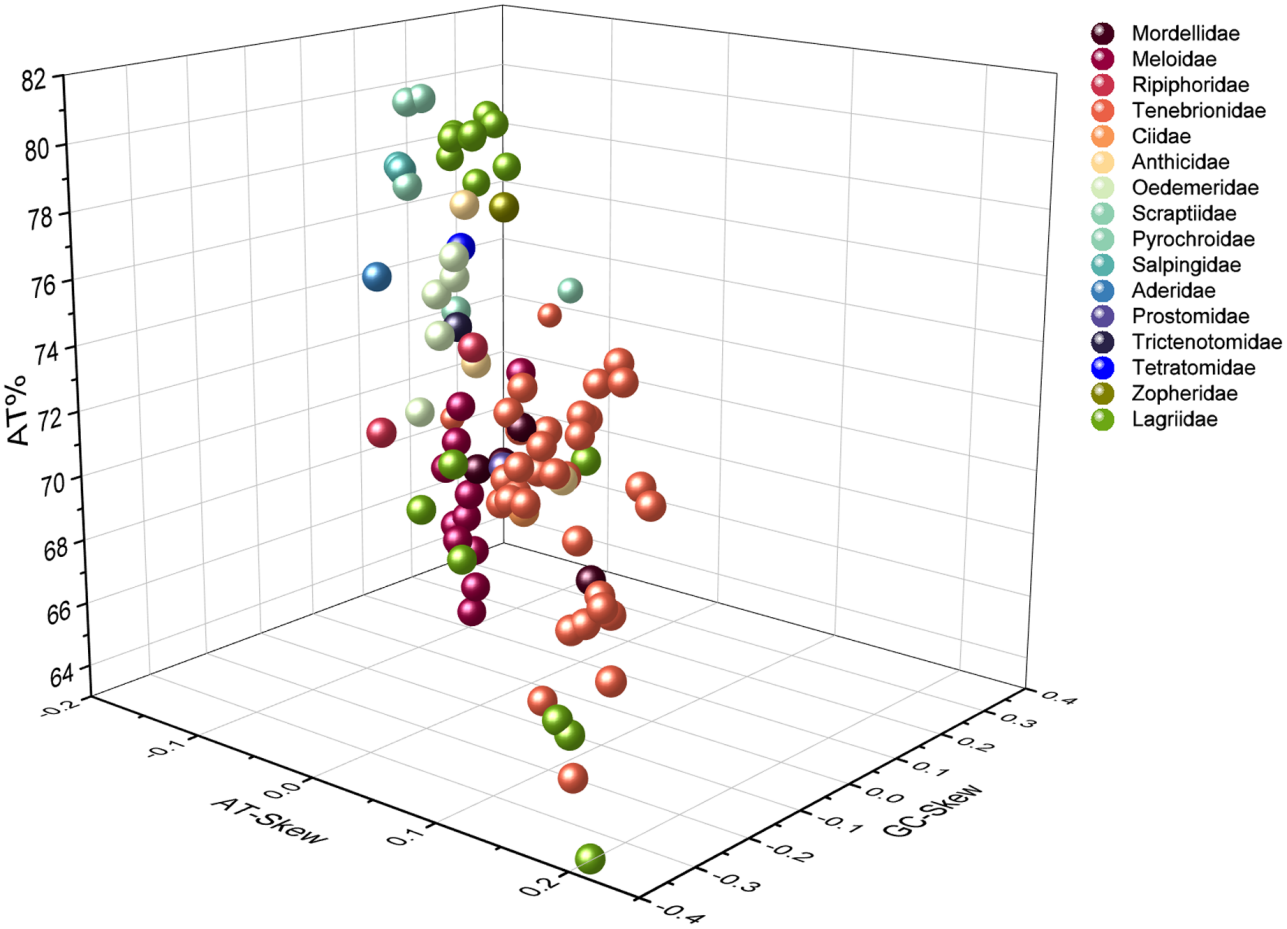
Three‐dimensional scatter plot of the AT‐skew, GC‐skew, and A + T content from 90 mtgenomes in Tenebrionoidea. The different colors of figures represent the different families of mtgenomes.

We completely sequenced mitogenomes from 19 species belonging to Tenebrionoidea in the present study (accession numbers in Table 1), with 11 of them being reported for the first time, all within Lagriidae. The complete mtgenomes from 82 species investigated contain the typical 37 genes (including 13 PCGs, 2 rRNAs, and 22 tRNAs) and one control region (CR). There are 22 genes (nine PCGs and 13 tRNAs) located on the majority coding strand (J‐strand), whereas the other 15 genes (four PCGs, nine tRNAs, and two rRNAs) are on the minority strand (N‐strand; Figure 1). These mtgenome sequences range in length from 14,777 bp (*Cerogria kikuchii*) to 16,861 bp (*Heterotarsus carinula*) with an average of 15,763 bp, and the length variation mainly results from the control region, intergenic overlap, and spacers. They all display obvious AT bias with A + T content ranging from 62.7% (*Casnonidea terminata*) to 81.6% (*Pyrochroidae* sp.) and an average of 73.6%. AT‐skew values range from −0.141 (*Paramarygmus* sp.) to 0.219 (*Strongylium pinfaense*) and GC‐skew from −0.375 (*S. pinfaense*) to 0.366 (*Paramarygmus* sp.; Figure 2).

The tRNAs sizes range from 57 to 82 bp, and all of the tRNAs can be folded into a typical clover‐leaf structure except for *tRNA*‐*Ser* (AGN) in which the dihydrouridine (DHU) arm is absent, and a UCU anticodon is present (Figure S1). The most frequently occurred base mismatches are U‐G, U‐U, and A‐G, and the mismatch A‐G is only occurred in *tRNA‐Trp*. For rRNAs, *rrnL* is located between the *trnL1* and *trnV*, ranging from 750 bp (*Anthicidae* sp.) to 1323 bp (*Alcidodes juglans*). The length of the *rrnS* ranges from 740 bp (*Cerogira popularis*) to 1269 bp (*Uloma* sp.), which is located between the *trnV* and the CR region. The percentage of AT content in rRNAs is 68.3%–84.3%. The CRs are located between the *rrnS* and *trnI*, and the percentage of AT content in this region is 72.2%–96.1%.

### 3.2 Rearrangement events

**FIGURE 3:**
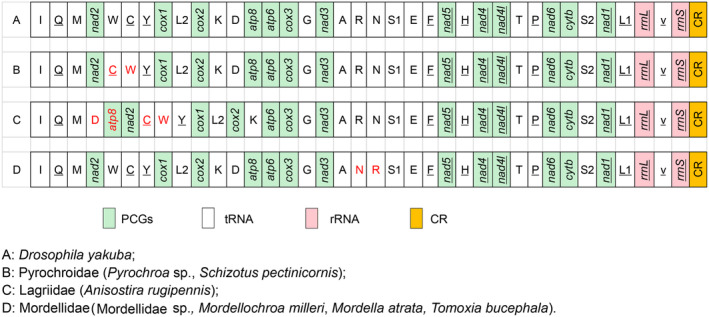
Gene rearrangement events in the 90 Tenebrionoidea mtgenomes. The underlined symbols are located on the N‐strand and others on the J‐strand. The green, white, pink, and orange blocks denote PCGs, tRNA, rRNA, and control regions, respectively. The red font means rearranged genes.

By comparing the composition and structure of the mtgenomes of these 90 Tenebrionoidea species, a total of seven Tenebrionoidea species were found to have gene rearrangement events. These rearrangement events occur in the three different families Lagriidae, Mordellidae, and Pyrochroidae (Figure 3). In other families, the gene order of the mtgenome is exactly the same as that of *drosophilid*. The tRNA genes have the highest frequency of rearrangement (*trnW‐trnC‐trnY* and *trnA‐trnR‐trnN‐trnS‐trnE‐trnF* gene cluster), followed by protein‐coding genes. The first one is found in the *trnW‐trnC‐trnY* gene cluster, and shuffling of *trnW* gene and *trnC* gene occurs in three species of *Schizotus pectinicornis* (Pyrochroidae), *Pyrochroidae* sp. (Pyrochroidae), and *Anisostira rugipennis* (Lagriidae). The second one in the *A. rugipennis* of Lagriidae, *trnD* and *ATP8* transposition to the upstream of *NAD2*, which is the first rearrangement event of protein‐coding genes found in the family Lagriidae. The last one is found in the *trnA‐trnR‐trnN‐trnS‐trnE‐trnF* gene cluster, and shuffling of *trnR* gene and *trnN* gene occurs in four species of the family Mordellidae: *Mordellidae* sp., *Mordellochroa milleri*, *Mordella atrata*, and *Tomoxia bucephala*.

### 3.3 Codon usage of PCGs and gene selection pressure

**FIGURE 4:**
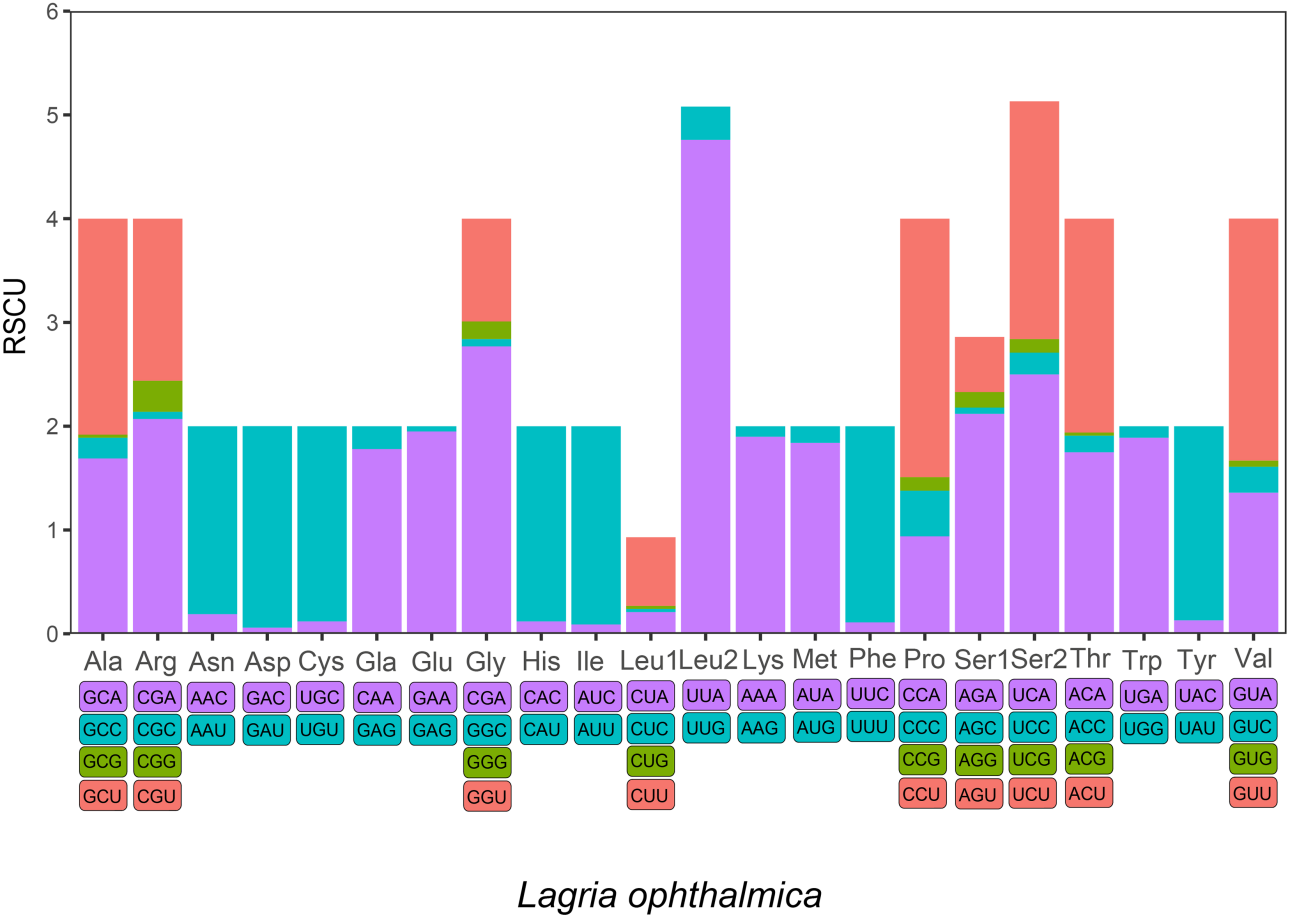
Relative synonymous codon usage (RSCU) analysis of each amino acid of *Lagria ophthalmica* (Lagriidae) mtgenomes in Tenebrionoidea. Codon families are provided on the x‐axis, and the frequency of codon usage is plotted on the y‐axis.

**FIGURE 5:**
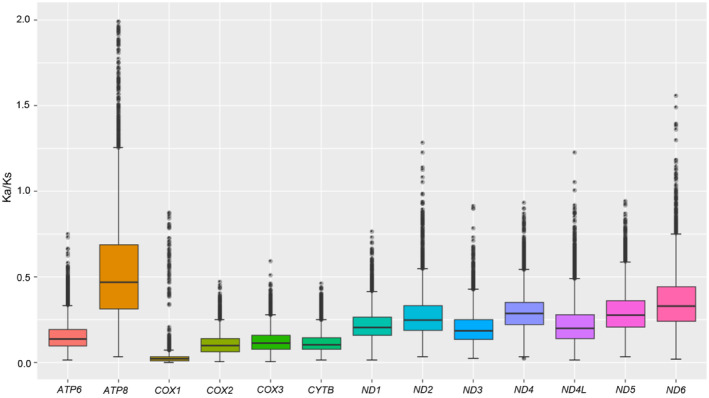
The evolutionary rates of 13 protein‐coding genes in the 90 Tenebrionoidea mtgenomes. Ka/Ks: The ratio of nonsynonymous nucleotide substitutions to synonymous nucleotide substitution. Neutral evolution (Ka/Ks = 1), purify selection (Ka/Ks < 1), and positive selection (Ka/Ks > 1).

Total PCGs nucleotide length ranges from 10,848 to 11,142 bp, and the AT contents range from 60.7% to 81.0%. Most of the PCGs initiate with the typical start codon ATN and TTG, whereas the special start codons AAC, AAT, AAA, and TCA are found for *COX1*; AAA for *COX2*; GTG for *ND1* and *ND4L*; AGG and AAA for *ND2* and GTG for *ND4*. The most frequently used stop codons are TAA and TAG, followed by the incomplete stop codons T and TA. The most frequently used codons are UUA (Leu2), UCU and UCA (Ser2), CGA (Arg), whereas AGC (Ser1), ACG (Thr), GCG (Ala), and CUG (Leu1) are the least used (Figure 4). For each PCG, the Ka/Ks ratio is less than one, and *ATP8* has the highest Ka/Ks ratio (0.33–0.67), followed by seven genes (*ND6*, *ND5*, *ND4*, *ND2*, *ND4L*, *ND1*, and *ND3*) with Ka/Ks ratios of 0.17–0.42. Complex IV (*COX1*, *COX2*, and *COX3*), Complex III (*CYTB*), and *ATP6* have low Ka/Ks ratios with a range from 0.01 to 0.17 (Figure 5). These results imply that all these 13 PCGs experienced purifying selection, especially Complex IV and Complex III.

### 3.4 Phylogenetic relationships

**FIGURE 6:**
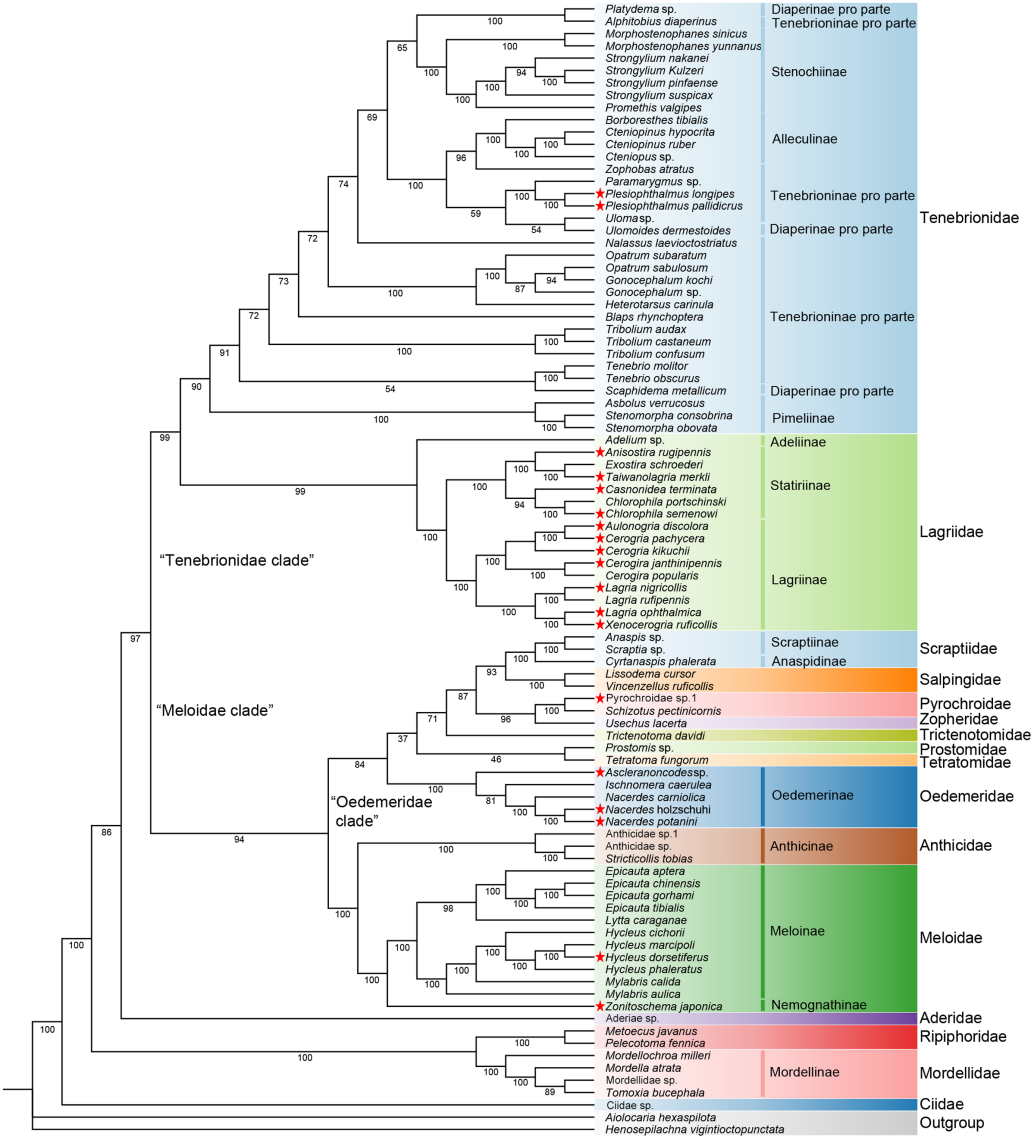
Mtgenome‐based phylogenetic relationships of 90 Tenebrionoidea species. They are constructed based on the amino acid (AA) data set using maximum likelihood methods. The different colors of species name blocks represent the different families. The red stars demonstrate the 19 newly sequenced mtgenomes in Tenebrionoidea in this study. Species and NCBI accession numbers for mtgenomes used in the phylogenetic analysis are provided in Table 1.

**FIGURE 7:**
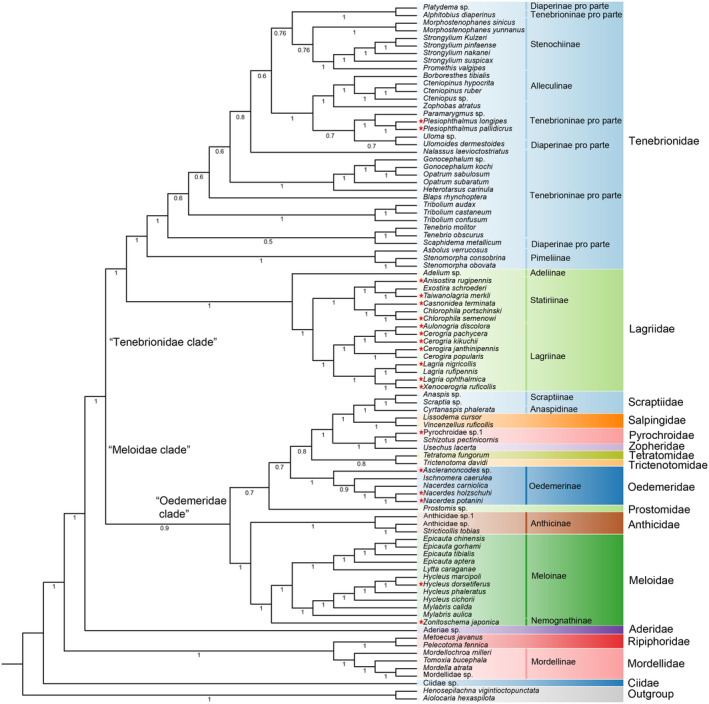
Mtgenome‐based phylogenetic relationships of 90 Tenebrionoidea species. They are constructed based on the amino acid (AA) data set using Bayesian inference methods.

Substitution saturation tests show no saturation for three data sets AA, PCG12, and PCG12 + rRNAs (Iss < Iss.cSym or Iss.cAsym, *p* < .05; Table 2), which proposes that these three data sets be appropriate for phylogenetic construction based on ML and BI. Six trees generated using these three data sets and both ML and BI are slightly different in topology (Figures 6, 7 and S3–S6). The Ciidae is located at the base of all phylogenetic trees, followed by families Mordellidae + Ripiphoridae. The Mordellidae was recovered monophyletic (PP = 1; BP = 100), and Ripiphoridae is close to it. The family Aderidae was suggested to be close to the “Meloidae clade” + “Tenebrionidae clade” branch. Both “Tenebrionidae clade” and “Meloidae clade” recovered monophyletic with both of them being sister groups (Figure 6). In the “Meloidae clade,” the families Meloidae + Anthicidae were suggested to be monophyletic (PP = 1; BP = 100), and it appears sister to the “Oedemeridae clade,” and the Meloidae was recovered monophyletic (PP = 1; BP = 100). In the “Oedemeridae clade,” the Oedemeridae, Pyrochroidae, Salpingidae, and Scraptiidae seem monophyletic (PP = 1; BP = 100), and Salpingidae is the sister of Scraptiidae. In the “Tenebrionidae clade,” the family Lagriidae and Tenebrionidae are monophyletic (PP = 1; BP = 90–100), and both of them are sister groups of each other. In Lagriidae, the subfamily Adeliinae is based on the subfamilies Lagriinae and Statininae, both of which were recovered monophyletic (PP = 1; BP = 100) and are sister groups of each other. Due to the insufficient sampling of Ripiphoridae and Anthicidae, we will not discuss whether they are monophyletic here.

**TABLE 2:** Substitution saturation test results.

Data partition	Iss	Iss.cSym^a^	Psym^b^	Iss.cAsym^c^	Pasym^d^
AA	0.005	0.703	0.0000	0.401	0.0000
PCG12 + RNA	0.364	0.818	0.0000	0.572	0.0000
PCG12	0.232	0.813	0.0000	0.570	0.0000
PCG1	0.309	0.807	0.0000	0.548	0.0000
PCG2	0.160	0.807	0.0000	0.548	0.0000
PCG3	0.710	0.807	0.0000	0.548	0.0000

^a^ Index of substitution saturation assuming a symmetrical true tree.

^b^ Probability of significant difference between Iss and Iss.cSym (two‐tailed test).

^c^ Index of substitution saturation assuming an asymmetrical true tree.

^d^ Probability of significant difference between Iss and Iss.cAsym (two‐tailed test).

In Tenebrionidae, the subfamily Pimeliinae appears to be a monophyletic group (PP = 1; BP = 100) and is located at the base of Tenebrionidae. The Alleculinae and Stenochiinae were recovered monophyletic (PP = 1; BP = 100), and the subfamilies Tenebrioninae and Diaperinae appeared in polyphyletic groups.

Six trees were generated using AA, PCG12, and PCG12 + 2 rRNAs data sets, and both ML and BI are slightly different in topology. For AA data set, the topologies using ML and BI are different in the positions of Prostomidae. Prostomidae and Tetratomidae are clustered as one clade in ML tree, but not in BI tree. For PCG12 data set, two same topologies of trees from BI and ML differ from two topologies of AA data set in phylogenetic relationship of the “Oedemeridae clade.” For PCG12 + 2 rRNAs data set, the positions of major families are the same as the four topologies of AA and PCG12 data sets, with only a few differences in the “Oedemeridae clade.”

### 3.5 Divergence time

**FIGURE 8:**
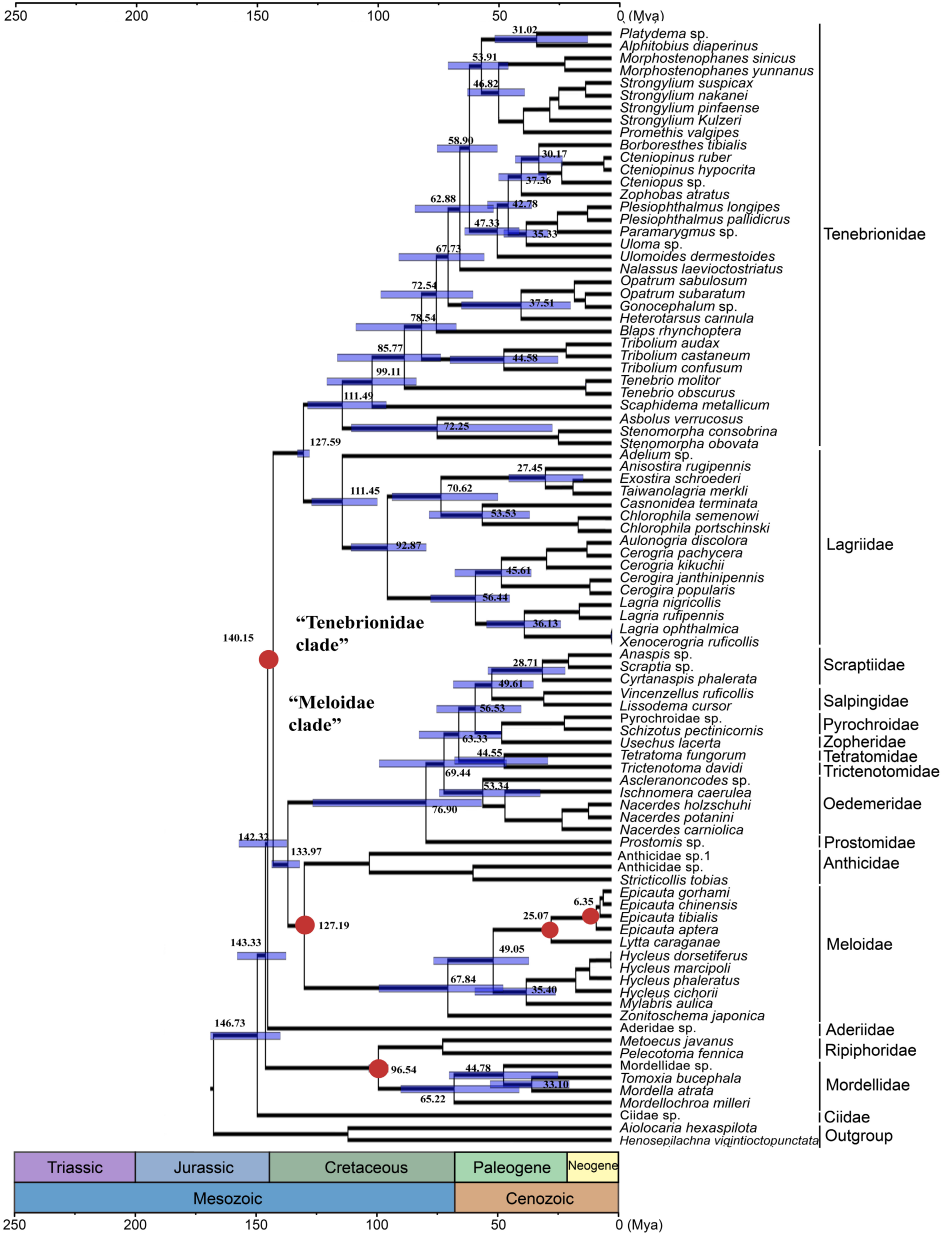
Timescale of Tenebrionoidea evolution displayed as a family‐level tree based on the amino acid data set. Ages were estimated based on three fossil calibration points (red dots). Blue bars indicate a 95% mean confidence interval (CI) of each node. A geological timescale is shown at the bottom.

The AA data set was used to estimate divergence time because AA had higher node support values than others in the initial phylogenetic assessment using Bayesian approach. Based on five fossil calibrations points of *Protoripidius burmiticus* (99.6–93.5 Mya), *Camelomorpha longicervix* (130.0–125.45 Mya), *Alphitopsis initialis* (130.0–125.45 Mya), *Meloe dominucanus* (20.44–13.82 Mya), and *Epicauta sanctoruensis* (5.33–3.6 Mya; filled red cycles in Figure 8), the superfamily Tenebrionoidea was inferred to originate in the late Jurassic (146.73 Mya, 95% confidence interval [CI]: 137.83–154.93 Mya), with most families subsequently diverging in the early Cretaceous (Figure 8). The family Mordellidae is the earliest diverged family in the superfamily and is estimated to originate at 69.97 Mya in the late Cretaceous. In the “Meloidae clade,” the family Meloidae is estimated to be derived at 67.84 Mya in the late Cretaceous, whereas the Oedemeridae at 53.34 Mya in the Paleogene. In the “Tenebrionidae clade,” the families Lagriidae and Tenebrionidae are estimated to originate at 111.45 and 111.49 Mya in Cretaceous, respectively. In the family Lagriidae, the subfamily Statiriinae diverged 70.62 Mya in the late Cretaceous, whereas the Lagriinae diverged 56.44 Mya in the early Paleogene. In the family Tenebrionidae, the subfamilies Pimeliinae, Alleculinae, and Stenochiinae originated at 72.25, 37.36, and 46.82 Mya between Cretaceous and Paleogene, respectively. All of these families/subfamilies are proposed to be monophyletic and confirmed in relationships in the phylogenetic analyses using different data or inferring methods, and others are not determined for their monophyly or relationships or have a few species included in the phylogenetic analyses, and therefore, they are not given an inferring of divergence time.

## 4 DISCUSSION

### 4.1 Characteristics of Tenebrionoidea mtgenomes

These 90 mtgenomes investigated in the present study in the superfamily Tenebrionoidea have a length variation from 14,777 to 16,861 bp, and the length variation mainly stems from CR, intergenic overlap, and spacers, which is consistent with earlier reports in Tenebrionoids (Burger et al., 2003). The nucleotide composition for all species exhibits obvious AT bias with high A + T content, similar to earlier reports in Tenebrionoids (Jie et al., 2016). All tRNA genes can form a complete clover secondary structure, except for *tRNA*‐*Ser* (AGN) which lacks the DHU arm, which seems to be a common feature of Tenebrionoidea (Song et al., 2018; Zhang et al., 2016). There are some rearrangement events in some species of the family Mordellidae, Lagriidae, and Pyrochroidae. The shuffling of the *trnR* and *trnN* genes (*trnA‐trnR‐trnN‐trnS‐trnE‐trnF* gene cluster) is found in all specials in the family Mordellidae; the translocation of the *trnD* and *ATP8* genes is found in the *A. rugipennis* of Lagriidae for the first time; the shuffling of the *trnC* and *trnW* genes (*trnW‐trnC‐trnY* gene cluster) is found in Pyrochroidae and Lagriidae. These gene rearrangements may be produced by abnormal priming of mitochondrial replication by a tRNA molecule to tandem duplications, which can provide an important reference for Tenebrionoidea phylogeny inference (Boore et al., 1998; Boore & Brown, 1998; Cameron, 2014; Timmermans & Vogler, 2012). The ATN and TTG are mainly used as the start codon, and TAA and TAG as the stop codon for the 13 PCGs, which is similar to other mtgenome sequences in Tenebrionoidea (Du et al., 2017). The Ka/Ks ratio is lower than one for all PCGs, which is consistent with earlier studies in Tenebrionoidea. The *COX1* gene has experienced strong evolutionary pressure in order to maintain its own functional requirements, whereas *ATP8* has experienced weak evolutionary pressures with allowing more mutations to accumulate in the mtgenome (Bai et al., 2018; Ou et al., 2016).

### 4.2 Overview of phylogenetic relationships

A total of 16 families are included in the phylogenetics and evolution analysis of the Tenebrionoidea, in which there are 10 families with at least two representative species included. The family Ciidae seems to be the earliest derived in these families, followed Mordellidae + Ripiphoridae, and Aderidae + “Meloidae clade” + “Tenebrionidae clade”. Ciidae was historily placed in the Cucujoidea (Crowson, 1955) and then to the superfamily Tenebrionoidea mainly based on characteristics of the aedeagus and the larval abdomen (Crowson, 1960). It was proposed to be a monophyly based on *18S* and *COX1* genes using ML and BI methods and demonstrated to be either a sister to Nitidulidae based on the reduced sample or at the base of the cucujoid‐tenebrionoid assemblage based on the entire sample (Buder et al., 2008). It was considered basal tenebrionoids based on 516 adult and larval morphological characteristics from 359 beetle taxa (Lawrence et al., 2011). The present study also suggests the family to be the basal tenebrionoids, but further investigation is necessary to elucidate its place with the inclusion of more species.

In this study, the “Mordellidae + Ripiphoridae” clade was proposed to be monophyletic, and the Mordellidae was also proposed to be monophyletic. The clade of Mordellidae + Ripiphoridae was proposed monophyletic in earlier molecular phylogeny inference based on five nuclear and mitochondrial genes with 300 genera in Tenebrionoidea using ML (Gunter et al., 2014). The Mordellidae was also proposed to be monophyletic in the study based on four molecular genes (*18S* rRNA, *28S* rRNA, *rrnL*, and *COX1*) with 128 species in Tenebrionoidea using ML (Batelka et al., 2016). Due to the insufficient sampling of Ripiphoridae, we will not discuss whether it is monophyletic here. However, it was proposed to be paraphyletic in the molecular phylogenetic study based on four mitochondrial and four nuclear gene fragments across 404 taxa (including 250 tenebrionid species) using ML (Kergoat, Soldati, et al., 2014).

Aderidae was proposed to be a monophyletic lineage in the earlier molecular phylogenetic study (Gunter et al., 2014). There is only one species in Aderidae to be included in the present study, which was close to the clade of “Meloidae clade” + “Tenebrionidae clade”, and its position and monophyletic status are yet to be resolved with more species involved. The “Meloidae clade” + “Tenebrionidae clade,” “Meloidae clade,” and “Tenebrionidae clade” are all proposed to be monophyletic, which are consistent with earlier studies based on five nuclear and mitochondrial genes with 300 genera in Tenebrionoidea using ML (Gunter et al., 2014) and eight mitochondrial and nuclear gene with 404 taxa in Coleoptera using ML (Kergoat, Soldati, et al., 2014).

### 4.3 Phylogenetic relationships of “Meloidae clade”

In the “Meloidae + Anthicidae” clade, Meloidae was proposed to be monophyletic in the present work, which supported an earlier study based on 4818 nuclear genes in 146 species of beetles using ML. In the present work, Anthicidae is identified as a sister group to Meloidae; however, in an earlier study, Meloidae was included within Anthicidae. Due to only three species of Anthicidae involved in the present work, the phylogeny of “Meloidae + Anthicidae” clade needs further investigation with the inclusion of more species and more molecular data.

In the “Oedemeridae clade”, the family Prostomidae seems to be located at the base of “Oedemeridae clade”, followed Oedemeridae and Trictenotomidae + Tetratomidae. Prostomidae was proposed a sister to the “pythid‐pyrochroid‐lineage” (including Trictenomatidae, Pyrochroidae, Salpingidae, and so on) based on morphology characteristics of the maxillary articulatory area, the abdominal tergite IX extending to the ventral side of the segment, and the strongly pronounced prognathous condition (Schunger et al., 2003). However, Prostomidae and Tetratomidae are clustered as one clade in ML tree, and the phylogenetic position of Prostomidae remained unresolved in the present analyses.

The family Oedemeridae is proposed to be monophyletic, which is consistent with the earlier phylogenetic study based on four gene (*16S* rRNA, *COX1*, *28S* and *18S* rRNA) sequences from 8441 taxa of Coleoptera, which removed misplaced single specimens and minor clades (Bocak et al., 2014). However, it was proposed to be paraphyletic in the phylogenetic study based on mitochondrial and nuclear genes (Gunter et al., 2014; Zhang et al., 2018), which suggests that the monophyletic status of the Oedemeridae need be further determined with more species included. Trictenotomidae and Tetratomidae are clustered as one clade using BI in this study, whereas Trictenotomidae was a sister to Boridae in earlier phylogenetic studies based on nuclear genes. There is only species in Trictenotomidae and Tetratomidae to be included in the present study, which suggests that the monophyletic status of the Trictenotomidae and Tetratomidae remains uncertain, and its position and monophyletic status are yet to be resolved with more species to involved.

Zopheridae and Pyrochroidae are clustered as one clade, and Pyrochroidae seems monophyletic in the present study. The monophyly of Pyrochroidae was also proposed in the earlier phylogenetic study based on 95 nuclear protein‐coding genes in 373 beetle species using ML and BI (Zhang et al., 2018) and in other phylogenetic studies based on mitochondrial and nuclear genes (Gunter et al., 2014; Kergoat, Soldati, et al., 2014). Zopheridae was a sister to Tetratomidae in earlier phylogenetic studies based on mitochondrial and nuclear genes (Kergoat, Soldati, et al., 2014), which suggests that the position and monophyletic status of Zopheridae and Pyrochroidae need to be further determined. The families Salpingidae and Scraptiidae seem monophyletic and are sister groups of each other in the present study. The monophyly of Salpingidae and Scraptiidae was also proposed in the earlier phylogenetic studies based on mitochondrial and nuclear genes (Bocak et al., 2014; Zhang et al., 2018), whereas Scraptiidae was proposed a paraphyletic group in other molecular phylogenetic studies (Hunt et al., 2007; Kergoat, Soldati, et al., 2014; Mckenna et al., 2015). The sister relationship of Salpingidae and Scraptiidae remains unclear due to the limited inclusion of only two or three species. Therefore, the position and monophyletic status of Salpingidae and Scraptiidae need to be further determined with more species included.

### 4.4 Phylogenetic relationships of “Tenebrionidae clade”

In the “Tenebrionidae clade,” the family Lagriidae and Tenebrionidae are monophyletic, and both of them are sister groups each other in the present study. The classification of Lagriidae has been debated over the years, and some scholars argue that Lagriidae should be classified as a subfamily within Tenebrionidae. However, the beetles of Lagriidae are leafivorous‐like beetles of Chrysomelidae, and the morphological characteristics of Lagriidae adapt much more for free‐moving and leaf‐feeding than the beetles from other subfamilies in Tenebrionidae. The monophyly of Lagriidae was proposed in the earlier morphology study based on the characteristics of abdominal defense glands, female reproductive tract, mouthparts morphology, and structure of the wings in Lagriid and Tenebrionoid (Doyen & Tschinkel, 1982). The monophyly was also proposed in earlier phylogenetic inference based on mtgenomes and nuclear genes (Gunter et al., 2014; Kergoat, Soldati, et al., 2014). The mtgenome‐based phylogeny of 36 species in Tenebrionidae suggested the monophyly of Lagriidae and Tenebrionidae and their sister relationships based on PCG123 data sets using BI and ML (Wu et al., 2021), which is consistent with the present study. The monophyly of Tenebrionidae was also proposed in some earlier phylogenetic studies based on mitochondrial and nuclear genes (Hunt et al., 2007; Timmermans et al., 2015).

The present study suggests the phylogenetic relationships of Adeliinae + (Lagriinae + Statininae) in the family Lagriidae and the monophyly of Lagriinae and Statininae. The monophyly of Lagriinae was proposed in earlier phylogenetic studies based on the morphology characteristics (Doyen, 1989) and also based on mitochondrial and nuclear genes (Gunter et al., 2014; Wu et al., 2021). The present study supports the monophyly of the subfamily Statiriinae for the first time. The Adeliinae is at the base of the family Lagriidae in this study, which is consistent with the earlier mtgenome‐based study (Wu et al., 2021). The monophyly of Adeliinae in Lagriidae is not yet determined due to only one species to be included.

In the family Tenebrionidae, the present study supports the monophyly of the subfamily Pimeliinae, Stenochiinae, and Alleculinae, whereas Tenebrioninae and Diaperinae are recognized as polyphyly. The subfamily Pimeliinae was also proposed to be monophyletic in earlier studies based on nuclear genes and mitochondrial genes (Gunter et al., 2014; Wu et al., 2021). The present study supports the monophyly of the subfamilies Stenochiinae and Alleculinae in Tenebrionidae, which was also proposed in earlier phylogenetic studies based on mitochondrial and nuclear genes (Kergoat, Soldati, et al., 2014; Wu et al., 2021). The subfamily Tenebrioninae and Diaperinae in these molecular‐based studies are found to be polyphyletic, which ineeds to be elucidated with more species involved.

### 4.5 Evolution of Tenebrionoidea

The Tenebrionoidea is inferred to originate in the late Jurassic (146.73 Mya, CI: 137.83–154.93 Mya) based on the mtgenomes and fossil calibrations points in the present study, which is consistent with earlier evolution studies in Coleoptera based on mitochondrial and nuclear genes (Cai et al., 2022; Mckenna et al., 2015; Zhang et al., 2016). The present results suggest that most families subsequently diverged in the Cretaceous. Angiosperms replaced the previously dominant gymnosperms during the Cretaceous, and the warm and humid environment had been produced in the Cretaceous, which provided food and habitat for the families in Tenebrionoidea. The family Ciidae seems to be the earliest derived in these families in the present study, which is inconsistent with earlier evolution studies (Kergoat, Bouchard, et al., 2014). The divergence time of Ciidae is not yet determined because only one species being included. The families Mordellidae and Ripiphoridae are among the earliest diverged families in the superfamily, Mordellidae is estimated to originate at 65.22 Mya in the late Cretaceous, which is consistent with the previous evolution study result based on 95 nuclear protein‐coding genes in 373 beetle species using ML and BI (Zhang et al., 2018). In the “Meloidae clade,” the families Meloidae (67.84 Mya) and Oedemeridae (53.34 Mya) originated in the late Cretaceous and early Paleogene, which is consistent with earlier evolution studies based on mitochondrial and nuclear genes (Kergoat, Bouchard, et al., 2014; López‐Estrada, 2019, 2022; Misof et al., 2014). In the “Tenebrionidae clade,” the family Lagriidae (111.45 Mya) was proposed to be derived in the middle Cretaceous, which is similar to the evolution study based on mitochondrial and nuclear genes in 404 beetle species (Kergoat, Bouchard, et al., 2014). The family Tenebrionidae (111.49 Mya) was suggested to be derived in the Cretaceous, which is consistent with the results of the previous evolution study based on 4818 nuclear genes.

In the family Lagriidae, the subfamilies Lagriinae (56.44 Mya) and Statiriinae (70.62 Mya) are proposed to be derived in the early Paleogene and late Cretaceous for the first time. In the family Tenebrionidae, the present study suggests the origin of the subfamilies Alleculinae (37.36 Mya) and Stenochiinae (46.82 Mya) in the Paleogene but is inconsistent with the results of the earlier evolution study (Kergoat, Bouchard, et al., 2014), which be due to differences in the taxa included, fossils constraints, and analysis methods applied. In further research, more accurate estimates of divergence times are necessary along with more precise fossil records for calibration and more complete sampling.

## 5 CONCLUSION

This is the first comprehensive study on the mtgenomes characteristics and mtgenome‐based phylogenetics in Tenebrionoidea. In the present study, we completely sequenced mitogenomes from 19 species belonging to Tenebrionoidea. The comprehensive analysis of 90 mtgenome sequences in Tenebrionoidea suggests that the AT‐skew, length variation, and codon usage are consistent with other reported mtgenomes in Tenebrionoidea. The families Mordellidae, Meloidae, Oedemeridae, Pyrochroidae, Salpingidae, Scraptiidae, Lagriidae, and Tenebrionidae were suggested to be monophyletic. Ciidae is at the base of the superfamily of Tenebrionoidea, and the Mordellidae is close to the Ripiphoridae. The “Tenebrionidae clade” and “Meloidae clade” are monophyletic, and both of them are sister groups. In the “Meloidae clade,” Meloidae is close to Anthicidae. In the “Tenebrionidae clade,” the family Lagriidae and Tenebrionidae are sister groups. In Lagriidae, the subfamily Adeliinae is based on the subfamilies Lagriinae + Statininae. In Tenebrionidae, the subfamilies Pimeliinae, Alleculinae, and Stenochiinae were recovered monophyletic, and Tenebrioninae and Diaperinae are polyphyletic. The divergence time analysis suggests that Tenebrionoidea originated in the late Jurassic, Meloidae Mordellidae, Lagriidae, and Tenebrionidae in the Cretaceous, Oedemeridae in Paleogene.

## AUTHOR CONTRIBUTIONS

**Yun‐Jian Hu:** Conceptualization (equal); software (equal); supervision (equal); validation (equal); writing – original draft (equal). **Feng‐Fan Jia:** Data curation (equal); software (equal); visualization (equal). **Li Hu:** Software (equal); visualization (equal); writing – original draft (equal). **Chuan Wu:** Investigation (equal); supervision (equal). **Tian Tian:** Investigation (equal); methodology (equal). **Ting‐Jing Li:** Conceptualization (equal); methodology (equal). **Bin Chen:** Conceptualization (equal); investigation (equal); methodology (equal); project administration (equal); resources (equal); software (equal); supervision (equal); validation (equal); writing – review and editing (equal).

## FUNDING INFORMATION

This research was supported by the National Natural Science Foundation of China (31872262, 31672363) and the National Key Program of Science and Technology Foundation Work of China (2015FY210300).

## CONFLICT OF INTEREST STATEMENT

The authors declare that they have no competing interests.

## Supporting information


Figure S1.

Figure S2.

Figure S3.

Figure S4.

Figure S5.

Figure S6.

Table S1.

Table S2.

Table S3.

Data S1.

## Data Availability

All data are available as tables and figures in the main paper and its supplementary files. The GenBank accession numbers for the 19 mtgenomes generated in the present study with detailed information in Table S1.
