# Supplementary figures and images for: Comparative mitogenome research revealed the phylogenetics and evolution of the superfamily Tenebrionoidea (Coleoptera: Polyphage)
Source: Ecol Evol. 2024 Jun 25;14(6):e11520. doi: 10.1002/ece3.11520 (PMC11199344; doi:10.1002/ece3.11520)

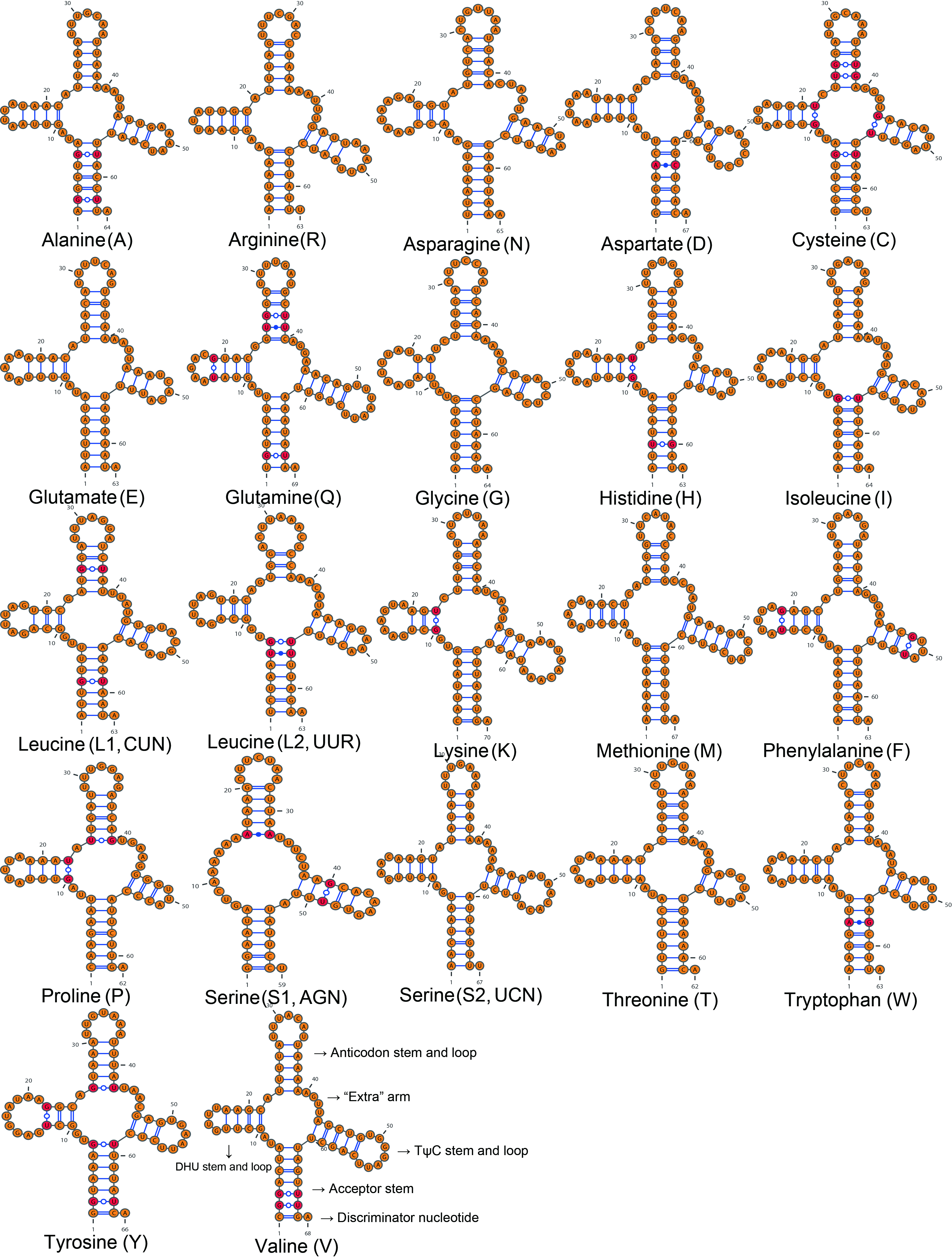

Supplement: Supplementary file 1 — Figure S1. [file ECE3-14-e11520-s009.tif]

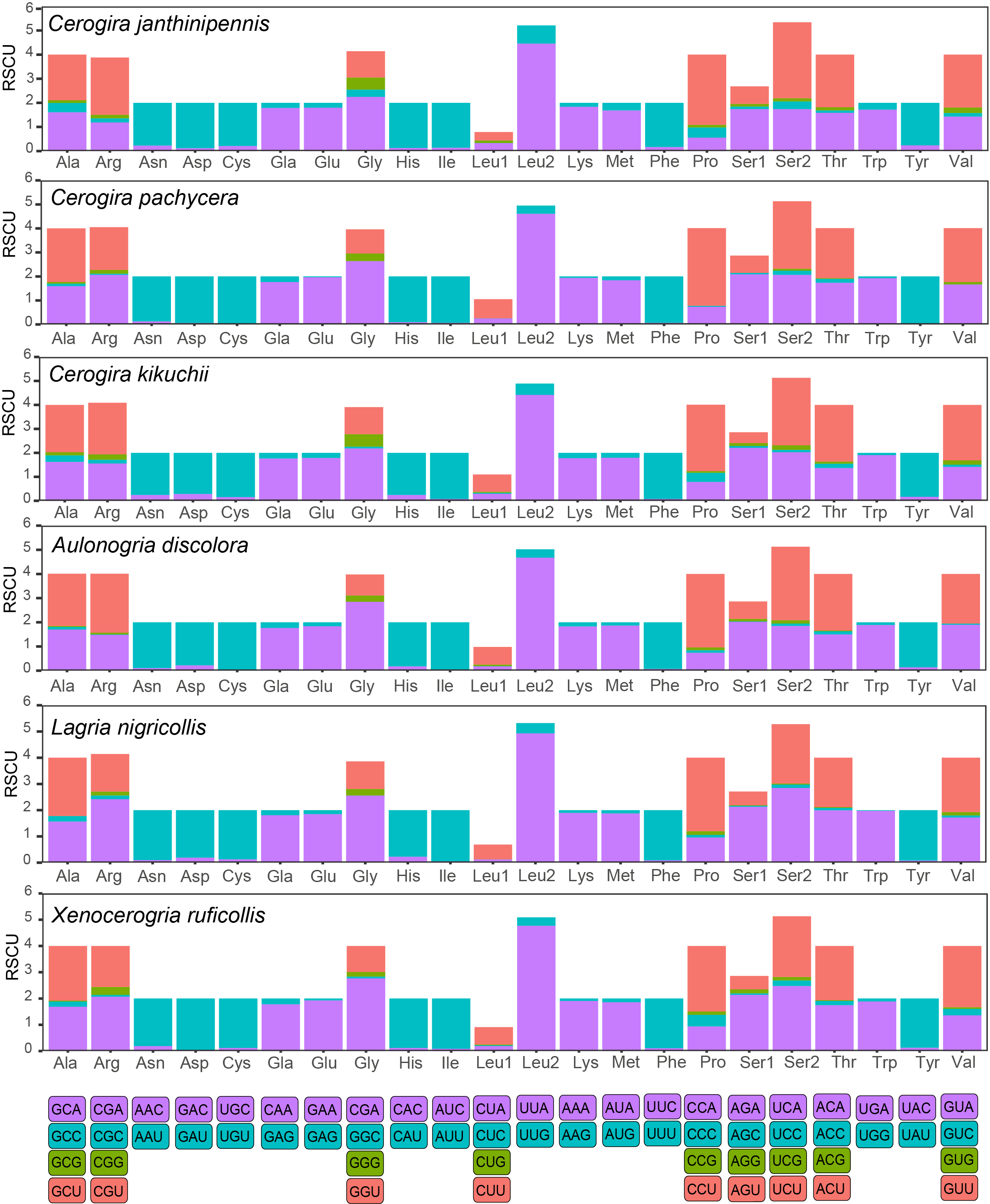

Supplement: Supplementary file 2 — Figure S2. [file ECE3-14-e11520-s007.tif]

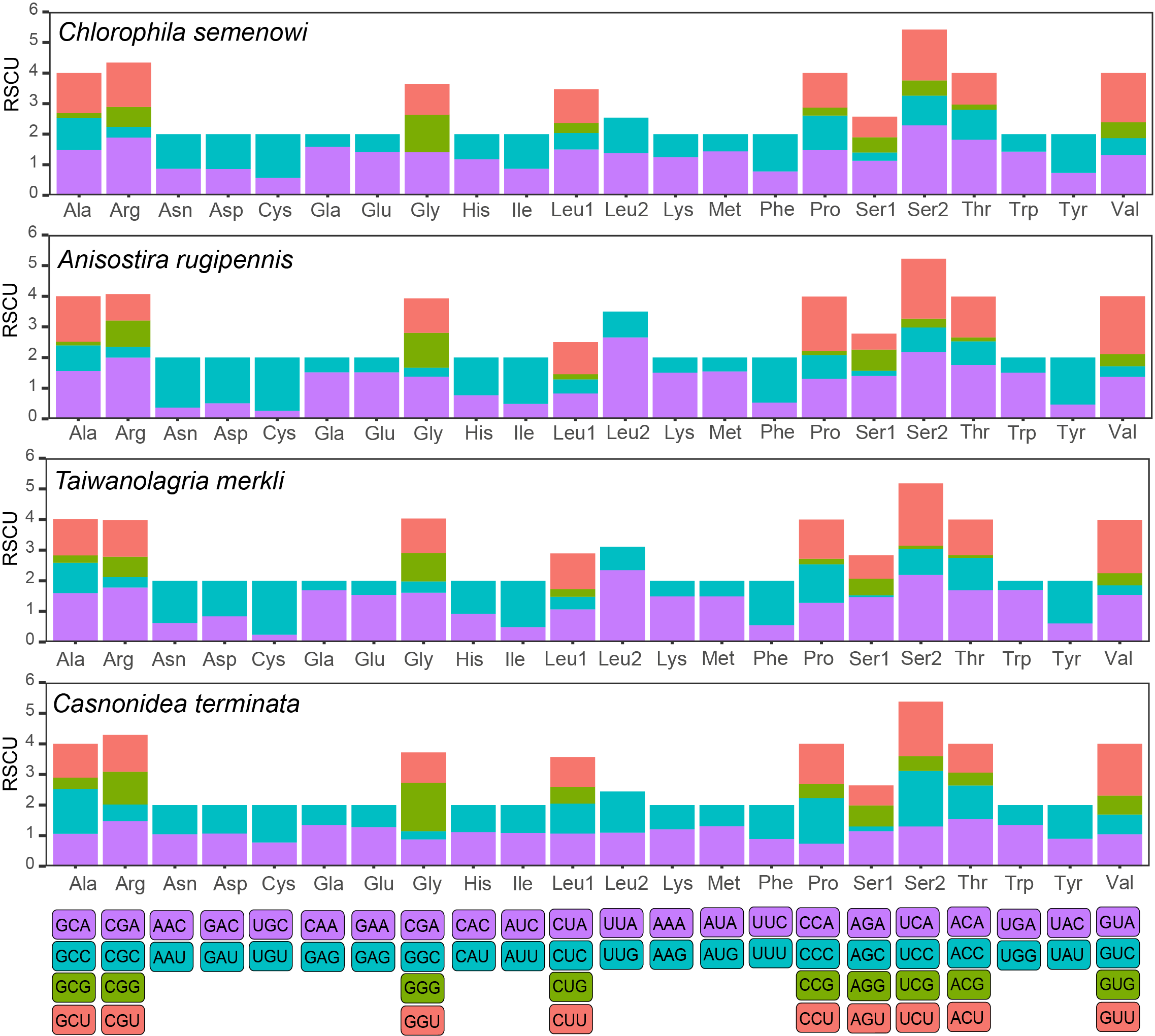

Supplement: Supplementary file 3 — Figure S3. [file ECE3-14-e11520-s002.tif]

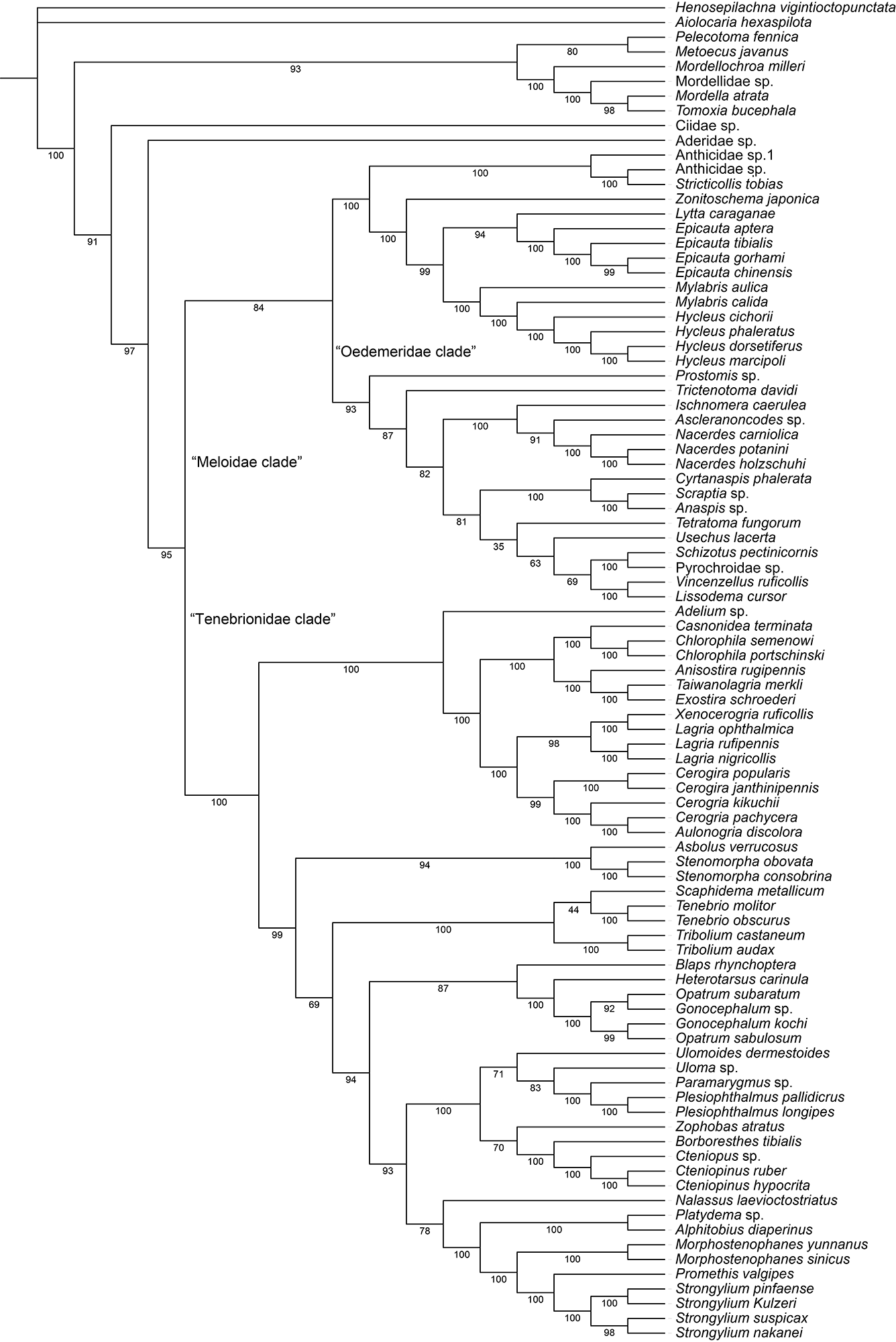

Supplement: Supplementary file 4 — Figure S4. [file ECE3-14-e11520-s004.tif]

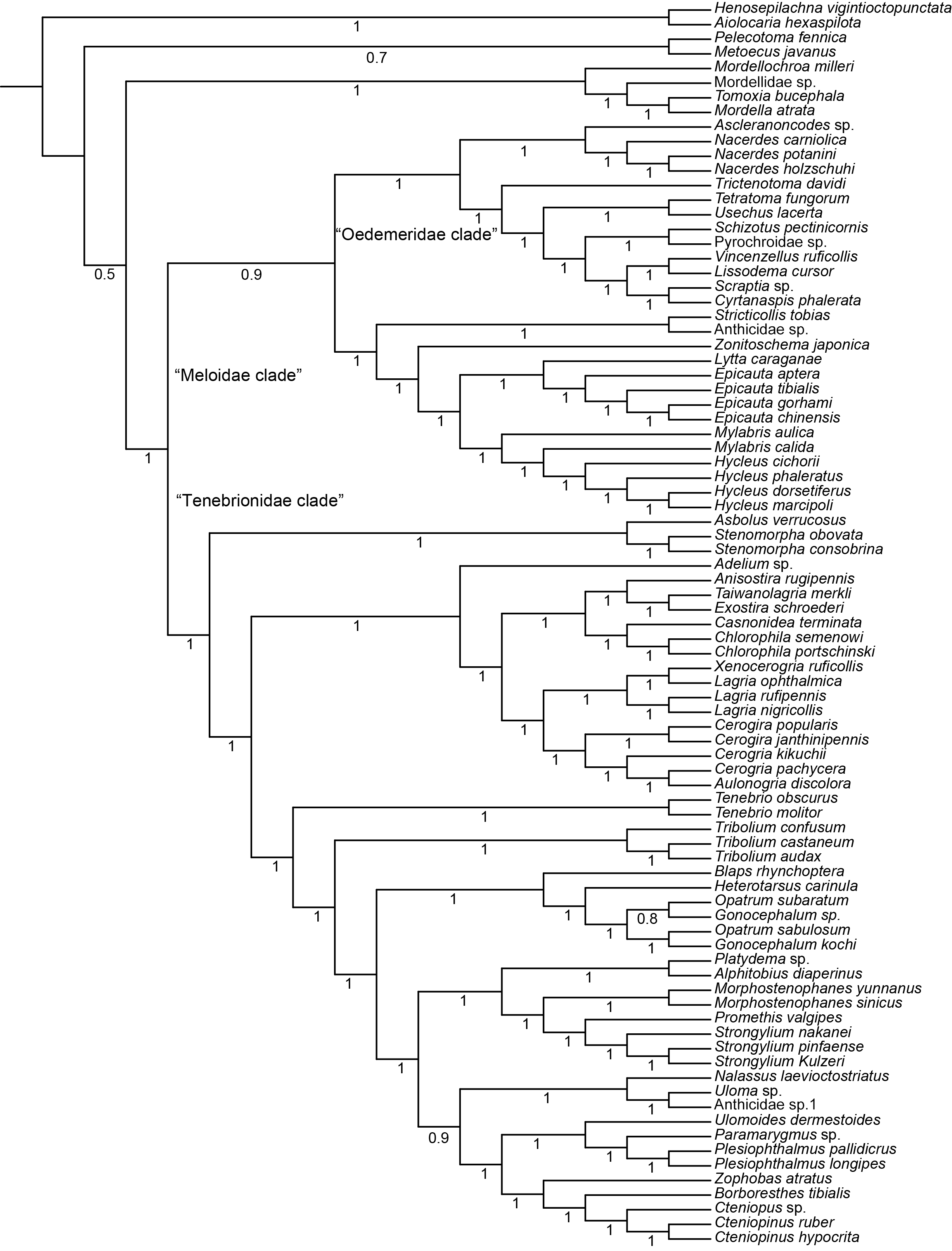

Supplement: Supplementary file 5 — Figure S5. [file ECE3-14-e11520-s006.tif]

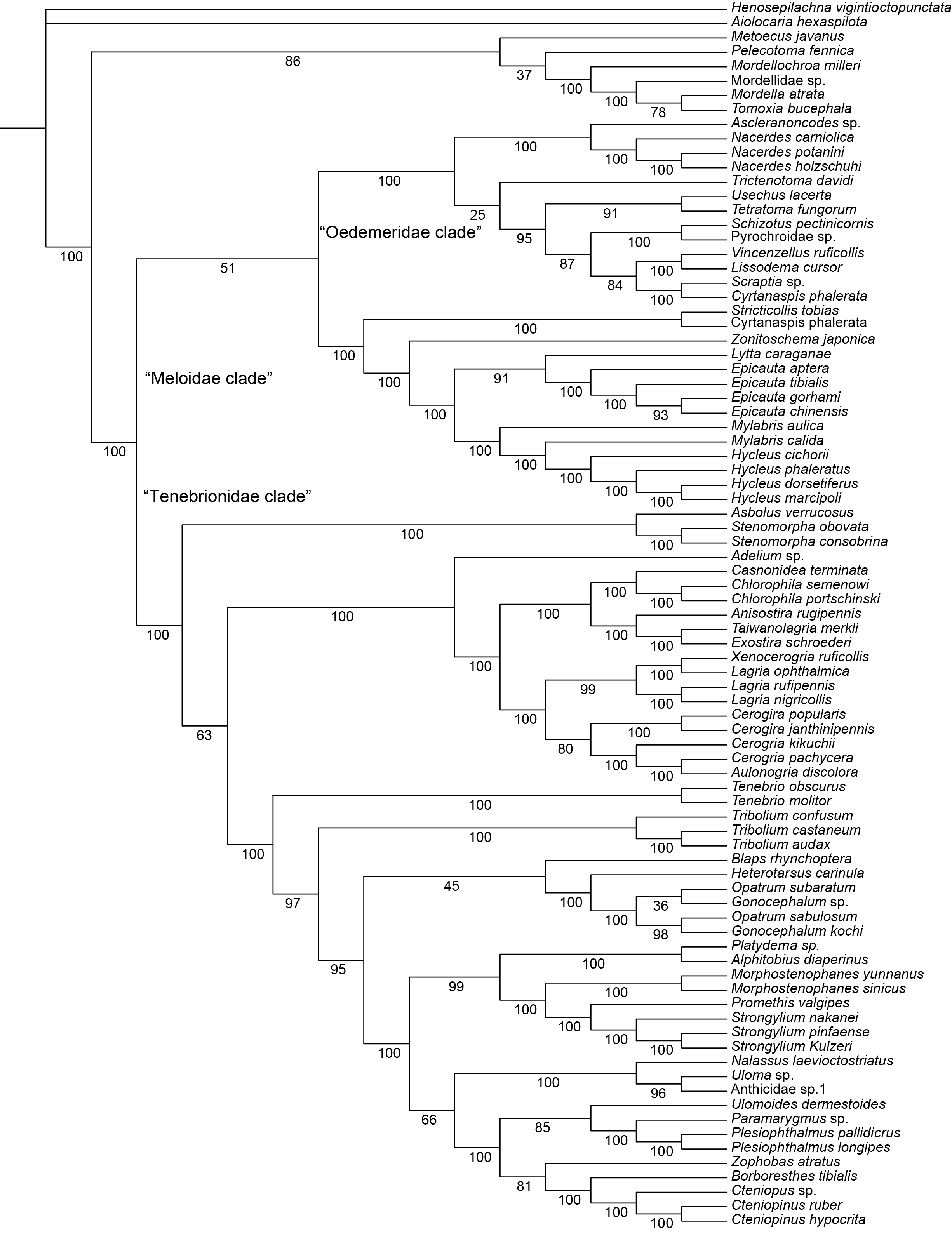

Supplement: Supplementary file 6 — Figure S6. [file ECE3-14-e11520-s005.tif]
