# Supplementary material for: Comparative mitogenome research revealed the phylogenetics and evolution of the superfamily Tenebrionoidea (Coleoptera: Polyphage)
Source: Ecol Evol. 2024 Jun 25;14(6):e11520. doi: 10.1002/ece3.11520 (PMC11199344; doi:10.1002/ece3.11520)
Supplement: Supplementary file 10 — Data S1. [file ECE3-14-e11520-s001.docx]

•Figures 1-8 Caption Text

Figures 1:
**Fig. 1.** Complete mtgenome genome structure of *Plesiophthalmus longipes* (A) and *Lagria nigricollis* (B). The species name and specific information regarding the genome (length, AT content, and the number of genes) are depicted in the center of the figures. The gene names are labeled on the outer circle, the green-, blue- and red- filled blocks indicate PCGs, tRNAs and rRNAs, respectively. The genes marked outward on the outer circle are located on the N-strand, whereas the genes inward located on the J- strand. L1, L2, S1 and S2 represent the *tRNA-Leu* (UAA), *tRNA-Leu* (UAG), *tRNA-Ser* (AGN) and *tRNA-Ser* (UCN), respectively.

Figures 2:

**Fig. 2.** Three-dimensional scatter plot of the AT-skew, GC-skew and A + T content from 90 mtgenomes in Tenebrionoidea. The different colors of figures represent the different families of mtgenomes.

Figures 3:
**Fig. 3.** Gene rearrangement events in the 90 Tenebrionoidea mtgenomes. The underlined symbols are located on the N-strand and others on the J-strand. The green, white, pink and orange blocks denote PCGs, tRNA, rRNA and control regions, respectively. The red font means rearranged genes.

Figures 4:

**Fig. 4.** Relative synonymous codon usage (RSCU) analysis of each amino acid of *Lagria ophthalmica* (Lagriidae) mtgenomes in Tenebrionoidea. Codon families are provided on the x-axis, frequency of codon usage is plotted on the y-axis.

Figures 5:

**Fig. 5.** The evolutionary rates of 13 protein-coding genes in the 90 Tenebrionoidea mtgenomes. Ka/Ks: The ratio of non-synonymous nucleotide substitutions to synonymous nucleotide substitution. Neutral evolution (Ka/Ks=1), Purify selection (Ka/Ks<1), Positive selection (Ka/Ks>1).

Figures 6:

**Fig. 6.** Mtgenome-based phylogenetic relationships of 90 Tenebrionoidea species. They are constructed based on amino acid (AA) dataset using Maximum likelihood methods. The different colors of species name blocks represent the different families. The red stars demonstrate the 19 newly sequenced mtgenomes in Tenebrionoidea in this study. Species and NCBI accession numbers for mtgenomes used in the phylogenetic analysis are provided in Table 1.

Figures 7:

**Fig. 7.** Mtgenome-based phylogenetic relationships of 90 Tenebrionoidea species. They are constructed based on amino acid (AA) dataset using Bayesian inference methods.

Figures 8:

**Fig. 8.** Timescale of Tenebrionoidea evolution displayed as a family-level tree based on the AA dataset. Ages were estimated based on three fossil calibration points (red dots). Blue bars indicate 95% mean confidence interval (CI) of each node. A geological timescale is shown at the bottom.

•Figures S1-S6 Caption Text

Figures S1:

The secondary structure of 22 transfer RNAs identified in the mtgenome of *Chlorophila semenowi* (Lagriidae). All *tRNA-Ser* (AGN) lack the DHU arm; bars denote Watson-Crick base pairings, and red dots denote mismatches base.

Figures S2:

Relative synonymous codon usage (RSCU) analysis of each amino acid of Lagriidae mtgenomes in Tenebrionoidea. Codon families are provided on the x-axis, frequency of codon usage is plotted on the y-axis.

Figures S3:

Phylogenetic relationships based on 1^st^ and 2^nd^ codon positions of PCGs (PCG12) dataset using Bayesian inference methods. The posterior probabilities for BI are shown on corresponding nodes in the trees. The GenBank accession numbers of the 90 mtgenome sequences are listed in Table 1.

Figures S4:
Phylogenetic relationships based on 1^st^ and 2^nd^ codon positions of PCGs (PCG12) dataset using Maximum likelihood methods.

Figures S5:

Phylogenetic relationships based on 13PCGs without 3rd site and 2 tRNAs (PCG12+rRNA) dataset using Bayesian inference methods.

Figures S6:
Phylogenetic relationships based on 13PCGs without 3rd site and 2 tRNAs (PCG12+rRNA) dataset using Maximum likelihood methods.
